# Relationship between Biomechanical Characteristics of Spinal Manipulation and Neural Responses in an Animal Model: Effect of Linear Control of Thrust Displacement versus Force, Thrust Amplitude, Thrust Duration, and Thrust Rate

**DOI:** 10.1155/2013/492039

**Published:** 2013-01-20

**Authors:** William R. Reed, Dong-Yuan Cao, Cynthia R. Long, Gregory N. Kawchuk, Joel G. Pickar

**Affiliations:** ^1^Palmer College of Chiropractic, Palmer Center for Chiropractic Research, Davenport, IA 52803, USA; ^2^Department of Physical Therapy, University of Alberta, Edmonton, AB, Canada T6G 2R3

## Abstract

High velocity low amplitude spinal manipulation (HVLA-SM) is used frequently to treat musculoskeletal complaints. Little is known about the intervention's biomechanical characteristics that determine its clinical benefit. Using an animal preparation, we determined how neural activity from lumbar muscle spindles during a lumbar HVLA-SM is affected by the type of thrust control and by the thrust's amplitude, duration, and rate. A mechanical device was used to apply a linear increase in thrust displacement or force and to control thrust duration. Under displacement control, neural responses during the HVLA-SM increased in a fashion graded with thrust amplitude. Under force control neural responses were similar regardless of the thrust amplitude. Decreasing thrust durations at all thrust amplitudes except the smallest thrust displacement had an overall significant effect on increasing muscle spindle activity during the HVLA-SMs. Under force control, spindle responses specifically and significantly increased between thrust durations of 75 and 150 ms suggesting the presence of a threshold value. Thrust velocities greater than 20–30 mm/s and thrust rates greater than 300 N/s tended to maximize the spindle responses. This study provides a basis for considering biomechanical characteristics of an HVLA-SM that should be measured and reported in clinical efficacy studies to help define effective clinical dosages.

## 1. Introduction

 Spinal manipulation is a form of manual therapy used frequently to address musculoskeletal complaints. Utilization data [1–3] indicates most patients receive a short lever, high-velocity low-amplitude type of spinal manipulation (HVLA-SM). An HVLA-SM has biomechanical characteristics broadly distinguished by a preload force that initially removes slack from the intervertebral tissues followed by a single thrust delivered quickly (on the order of tenths of a second or less). The thrust is often directed in a specific direction to an anatomical area of a pre-specified vertebra [4, 5]. A recent systematic review of randomized clinical trials specifically investigating the therapeutic benefit of HVLA-SM indicates that, despite the wide variability in how clinical outcomes have been measured and reported, HVLA-SM produces modest yet consistent clinical benefit [6]. The mechanisms of action are elusive.

Like other therapeutic interventions requiring manual deftness, such as surgery, the successful delivery of an HVLA-SM combines knowledge about the motor skills critical for maximizing clinical success and mastery of those motor skills [7]. These motor skills include learning to control the applied force or displacement during the manipulative thrust. With control of these parameters it is important to know whether it is more effective to control force versus displacement and whether there is a thrust amplitude or range of amplitudes critical to producing favorable clinical outcomes? Similarly, is there a specific thrust duration or range of durations over which the peak force or displacement amplitude develops that can make an HVLA-SM most effective? The answers to these questions may be viewed as elements of the manipulation's dosage, conceptually similar to chemical characteristics, such as molecular composition and permeability, which determine a drug's clinically effective dosage.

Evidence-based patient care informed by data from clinical studies requires knowing as precisely as possible the relevant characteristics of treatments used in these studies. To our knowledge, no clinical studies have yet addressed the relationship between an HVLA-SM's biomechanical characteristics and any clinical outcome. While the relationship between the number of HVLA-SM treatments and clinical benefit has been studied [8, 9], the manipulation's biomechanical characteristics were neither standardized nor measured. Consequently, how these characteristics might have affected the clinical outcomes is not determinable. Initial animal studies suggest that HVLA-SMs delivered with thrust durations 100 ms or less substantially increase sensory input from paraspinal proprioceptors [10, 11]. Thrust duration interacts with thrust amplitude toward changing spinal stiffness in ways that have only begun to be studied [12]. Some but not all neuromuscular responses from paraspinal muscles are graded with both the manipulation's amplitude and duration [13].

Motivated by the idea that our ability to determine an HVLA-SM's clinical efficacy is hampered by our lack of knowledge about the relationship between the intervention's biomechanical characteristics and clinical outcomes, we used an animal preparation to determine the relationship between a simulated HVLA-SM's biomechanical characteristics and changes in neural activity from muscle spindles in lumbar paraspinal muscles. This approach was used because across healthcare professions that use spinal manipulation, spinal manipulation's mechanism of action is thought to be largely mediated by changes in spinal biomechanics and/or changes in sensory input arising from paraspinal tissues [14–17] including muscle spindles in the back muscles [18]. Similar invasive studies could not be performed in humans. The purpose of the study was to determine how the type of thrust control (applied as a linear increase in either force or displacement), the thrust amplitude, thrust duration, and consequent thrust rate of an HVLA-SM affected the pattern or magnitude of neural activity from muscle spindles. Defining the physical characteristics of an HVLA-SM that have the greatest influence on neural activity will help clarify those elements that have the greatest potential to enhance the effectiveness of this intervention.

## 2. Methods

### 2.1. General Description

Data were obtained from single, peripheral sensory neurons innervating muscle spindles in multifidus or longissimus muscles in a large sample of anesthetized cats (*n* = 112) of either sex weighing an average of 3.97 kg (SD 0.85). All experiments were reviewed for ethical considerations and approved by Palmer College of Chiropractic Institutional Animal Care and Use Committee (no. 20070101). HVLA-SMs were delivered using a programmable, computer-controlled mechanical device enabling us to systematically control the manipulation's biomechanical characteristics. HVLA-SMs were considered to simulate a clinically delivered manipulation based upon using a range of thrust amplitudes and durations similar to those reported in the clinical literature (see Sections  2.6 and  2.7). Each HVLA-SM was applied to the cutaneous tissues overlying the L_6_ vertebra (cats have 7 vertebrae) while simultaneously recording neural action potentials from muscle spindles innervated by the L_6_ spinal nerve. The frequency of action potentials was determined before and during the delivery of each HVLA-SM.

Responses from only one neuron could be investigated per cat because, following the series of HVLA-SMs, cutaneous tissues overlying the L_6_ vertebra were cut to expose deeper back tissues in order to confirm that the neuron innervated a muscle spindle in the lumbar multifidus or longissimus muscle. No responses to HVLA-SM were studied once the cutaneous tissues overlying the L_6_ vertebra were cut. Calibrated nylon monofilaments (Stoelting, IL, USA) were applied to the exposed back muscles to verify the location of the most sensitive portion of the back from which the neuron could be activated (i.e., the neuron's receptive field). Sensory neurons were identified as muscle spindle neurons based upon standard neurophysiological techniques including their increased discharge to succinylcholine (100–400 mg/kg intra-arterially (ia)) and decreased discharge to electrically induced muscle contraction as described previously [19]. In addition, to help differentiate muscle spindle from Golgi Tendon Organ responses, we determined whether the neuron was able to produce a sustained response to a fast vibratory stimulus applied to the muscle's surface close to the neuron's receptive field [20].

### 2.2. General Surgery

Surgical procedures have been presented previously [19, 21, 22] and are also described here. Anesthesia was induced using a mixture of O_2_ and isoflurane, first delivered to a sealed plastic chamber (5 L/min and 5%, resp.), and then through a facemask (2 L/min and 2%). After placing catheters in a common carotid artery and an external jugular vein to monitor blood pressure and introduce fluids, respectively, and after intubating the trachea to mechanically ventilate the lungs, deep anesthesia was maintained with Nembutal (35 mg/kg intravenously (iv)). Additional doses (5 mg/kg, iv) were administered when the cat demonstrated a withdrawal reflex to noxious pinching of the toe pad, or when mean arterial pressure either increased spontaneously above 120 mmHg or in response to surgical manipulation. Arterial pH, PCO_2_, and PO_2_ were regularly monitored throughout the experiment using an i-Stat pH/blood gas analyzer (i-Stat Corp., East Windsor, NJ, USA) and maintained within the normal range (pH 7.32 to 7.43; PCO_2_, 32–37 mmHg; PO_2_, >85 mmHg).

### 2.3. Spinal Surgery and Nerve Preparation

**Figure 1:**
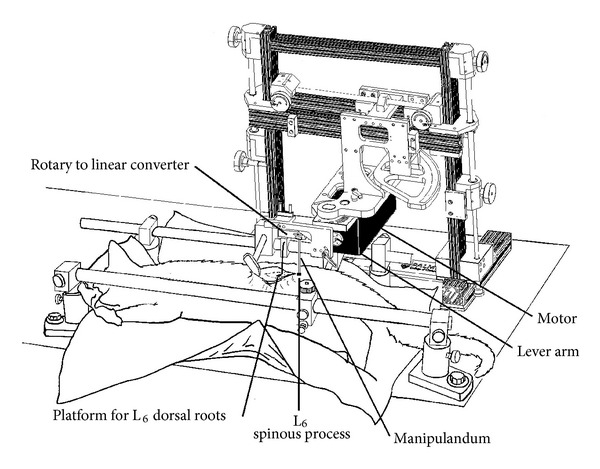
Schematic of the experimental set-up showing exposure of the L_6_ dorsal roots, the intact lower lumbar spine, and the device used to control delivery of the spinal manipulations at the L_6_ spinous process.

Studying the effects of a spinal manipulation on responses from peripheral sensory neurons innervating the manipulated back tissues is problematic because access to the nervous system is limited [19, 23]. Peripheral nerves innervating lumbar spinal tissues are not lengthy and substantial removal of the dorsal musculature appears necessary for accessing neural recording sites in the dorsal roots. We have previously developed [19] an *in vivo* cat preparation and have now improved upon it by keeping the skin and deep paraspinal tissues intact bilaterally from the L_6_ vertebra caudalwards where the HVLA-SM is delivered. The L_6_ lumbar dorsal roots are sufficiently exposed for electrophysiological recordings. The experimental setup is shown in Figure 1.

Exposing the L_6_ dorsal roots and keeping the lower lumbar spine intact takes advantage of the anatomical fact that the caudal most rootlets of L_6_ enter the spinal cord approximately two vertebral segments (30–35 mm) rostral to the L_6_ spinal nerve's entry through the L_6_ intervertebral foramina. Only the skin over the L_4_ and L_5_ vertebra was incised and the lumbodorsal fascia opened only from L_4_ to L_5_. Multifidus, longissimus, and lumbococcygeus muscles over only the L_4_ and L_5_ vertebrae on the left side were removed. The laminae of the L_5_ vertebra and of the caudal half of the L_4_ vertebra were removed to expose the cranial portion of the L_6_ dorsal rootlets. The lumbar spine was anchored at L_4_ and the pelvis by fixing the L_4_ spinous process and the iliac crests in a Kopf spinal unit (Figure 1). The paraspinal tissues were bathed in warm mineral oil (37°C) to prevent desiccation. With the dorsal roots exposed and placed on a glass platform (Figure 1), thin filaments from the rootlets were teased using forceps under a dissecting microscope until action potentials from a single neuron were identified. The action potentials were recorded using a PC based data acquisition system (Spike 2, Cambridge Electronic Design, UK).

### 2.4. Delivery of HVLA-SM

Components of the mechanical device that delivered and systematically controlled the manipulation's biomechanical characteristics are shown schematically in Figure 1. The device was comprised of an electronic feedback control system, a motor, and a lever arm attached to the motor's shaft (Aurora Scientific, Lever System Model 310). Computer-controlled rotation of the motor's shaft rotated the lever arm. The lever arm was attached to a custom built rotary-to-linear converter which in turn was attached to a manipulandum (see Figure 1) that contacted the back of the cat. The rotary-to-linear converter consisted of a polycarbonate block machined with a narrow slot that received the end of the motor's lever arm and held two parallel guide posts passing through linear bearings in an adjacent fixed bearing block. The manipulandum consisted of a thin titanium rod (0.2 cm diameter × 12 cm long) secured at one end into the rotary-to-linear converter and inserted at the other end into a small plexiglass tip. The tip made direct contact with skin overlying the L_6_ spinous process. The converter transformed the lever arm's rotary motion to linear motion of the manipulandum.

With the cat lying prone, HVLA-SMs were applied at the L_6_ spinous process in a vertical direction, that is, toward ventralward from the back of the cat. The electronic feedback control system allowed the motor to control either the force applied at the end of the lever arm (force control) or the distance traveled by the end of the lever arm (displacement control). The manipulandum was always positioned perpendicular to the lever arm so that force and displacement at the end of the lever arm were the same as at the back of the cat where it was contacted by the tip of the manipulandum. Forces and displacements during the HVLA-SM were simultaneously measured at outputs from the control system.

**Figure 2:**
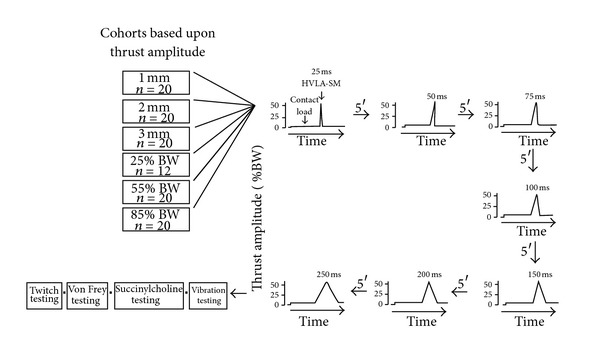
Schematic showing the experimental protocols. The protocol for a cat in the 55% body weight (BW) cohort is depicted. Contact load applied prior to the HVLA-SM removed slack from the soft tissues and engaged the L_6_ vertebra. The high-velocity low-amplitude spinal manipulations (HVLA-SM) were applied at the spinous process of the L_6_ vertebra. The 7 thrust durations were presented in random order. Duration of the contact load not drawn to scale. BW: body weight.

The mechanical profile (amplitude versus time) of a clinically delivered HVLA-SM can be roughly represented by the shape of an up-side down letter “V” [24–26]. The HVLA-SM's thrust phase is represented by the ascending arm of the “Λ” (see HVLA-SMs in Figure 2). As shown in Figure 2, the vertical height represents thrust amplitude (measured as applied force or displacement) and its horizontal length represents thrust duration (measured in milliseconds). Reaching the thrust amplitude was always controlled linearly that is, in force control the manipulative force increased at a constant rate, and in displacement control the manipulative displacement increased at a constant velocity.

An important goal with the experimental setup was to have physical contact between the cat's back and the manipulandum be similar to the physical contact between a clinician's hand and the lumbar spine of a patient. One way we did this was to have the manipulandum's tip make direct contact with the intact skin overlying the L_6_ spinous process as previously described. This improved upon earlier studies where the skin had been cut and toothed forceps clamped directly onto the spinous process [10, 11]. The second way was to customize the manipulandum's plexiglass tip by scaling its contact area with the skin to that used clinically in the lumbar spine. In the human, peak thrust forces are distributed over a relatively circular area between 350 and 1480 mm^2^ [27] when the pisiform bone is used to apply an HVLA-SM. We scaled this area but not its shape to the cat using a ratio of heights (from caudal to cranial tip of the articular processes) between the cat and human lumbar vertebra. We took direct measurements from cat and human lumbar specimens. The cat L_6_ vertebra is 23 mm in height and the comparable human vertebra (L_4_) is 43 mm. The 0.53 ratio was slightly reduced to 0.45. The final scaled surface area was 70 mm^2^. The tip's shape was rectangular (7 mm × 10 mm) with a narrow channel (5 mm wide × 2 mm deep) designed to cradle the sides of the spinous process and help prevent lateral slippage during the HVLA-SM.

### 2.5. Initial Contact Load

Clinically, the thrust of an HVLA-SM is intended to impart movement to a vertebra [4]. To ensure that vertebral movement could occur at the start of the thrust, we developed a method to identify the applied force which engaged the soft tissues and beyond which the L_6_ vertebra would be expected to move ventralward (contact load). In each cat, the manipulandum was placed over the L_6_ spinous process and slowly lowered in displacement control (1.33 mm/s), translating the contact point 4 mm ventralward. We recorded the displacement and the force required to achieve the displacement and plotted them as a force-displacement (F-D) curve. A regression line was fit to the curve's toe region. The force at which the F-D curve diverged from the regression line was considered the contact load reasoning that the level of force at the beginning of this stiffer region represented compression of and/or slack removal from adjacent soft tissues and engagement of vertebral movement. In 19 cats, we visually confirmed that movement had occurred with this contact load. This was accomplished by attaching a vertical post to the cranial edge of the L_6_ spinous process and capturing the physical movement using a high resolution optical recording system (Motion Pro Digital Image System, Redlake MASD Inc, CA, USA). Video capture was time synched with data acquisition of force and displacement. The force at which movement occurred was compared with the force at which divergence occurred. On average, physical movement of the vertebra began before contact load was attained on the F-D curve (60.8 (SD 14.9) gm versus 64.3 (12.7) gm, resp.).

### 2.6. Deciding upon Thrust Amplitudes

Choosing clinically relevant thrust amplitudes to use experimentally in an animal model is not straightforward. Clinical effects of spinal manipulation have been investigated in horses [13, 28] but the amplitudes used were not been reported. Human clinical studies have measured applied thrust forces but have not provided decision principles that guide the clinician's behavior. The range of forces used for treating the lumbosacral region is reported at 220–550 N [24, 29]. Whether this range reflects random variation around a mean value or a clinician's intuitive scaling factor is unknown. Nonetheless, we have standardized these forces based upon body weight (BW) assuming an average human BW of 70 kg. This yielded thrust forces in the lumbosacral region that ranged from 31% to 78% BW. We used 3 thrust forces (25%, 55%, and 85%) which encompassed the range used in humans.

Choosing a range of thrust displacements is not straightforward either. In the human cadaveric lumbar spine, Ianuzzi and Khalsa [30] simulated a side posture HVLA-SM using force-time characteristics described above. The manipulated vertebra translated approximately 1.5 ± 0.5 mm and rotated 2–3.5 ± 1.0° in the direction of the applied force. Nathan and Keller [31] measured intervertebral lumbar motion using pins inserted into human lumbar spinous processes using a mechanical adjusting device (Activator Adjusting Instrument [32]). This device delivers a force-time profile lower in amplitude (53 N), shorter in duration (17 ms), but with a faster force rate (3100 N/s) compared with a manually applied HVLA-SM. Using the device to deliver a spinal manipulation at the L_2_ spinous process produced 1.62 ± 1.06 mm peak axial displacements (in the longitudinal plane), 0.48 ± 0.1 mm shear displacements (in the transverse plane), and 0.89 ± 0.49° rotations between L_3_ and L_4_. Smith et al. [33] found that manipulations given with the device also evoked similar vertebral displacements in the lumbar spine of the dog. L_2_ translated 0.71 ± 0.03 mm and rotated 0.53 ± 0.15° on L_3_ with impulse loads of 53 N. Taken together, these data suggest that relative vertebral displacements are more than 0.5 mm but less than 2 mm. We used thrust displacements (1, 2, and 3 mm) that included and were slightly higher than this range.

### 2.7. Deciding upon Thrust Durations

A range of thrust durations that might be clinically relevant were used. In the cervical spine, the time to peak thrust amplitude ranges from 30 to 65 ms [34]. For HVLA-SM applied to the thoracic and lumbar regions, the thrust phase rises to a peak load in less than 150 ms [24–26]. We used a wide range of thrust durations (25, 50, 75, 100, 150, 200, and 250 ms) which encompassed those used clinically.

### 2.8. Experimental Design

The 112 cats were divided into 6 groups. Each group was considered a cohort because its members received the same controlled thrust magnitude. Cohorts were named according to the thrust magnitude they received (1 mm, 2 mm, 3 mm, 25% BW, 55% BW, and 85% BW cohorts). Each cat received the 7 thrust durations (25, 50, 75, 100, 150, 200, and 250 ms). The 7 HVLA-SMs were each separated by 5 minutes.

Figure 2 is a schematic showing the experimental protocols. With the device programmed to deliver the desired thrust amplitude, contact load was applied for 30 s followed by an HVLA-SM with a 25 ms thrust duration. Five minutes later contact load was again applied followed by a 50 ms thrust duration and so on. The 7 thrust durations were presented in random order yielding a randomized complete block experimental design.

### 2.9. Data Management

Neural activity arising from manipulation-induced activation of the muscle spindle was determined by comparing activity during 2 s immediately preceding each HVLA-SM (baseline) with that during the thrust phase of the HVLA-SM. All neural activity was first quantified as instantaneous frequency (IF) by taking the reciprocal of the time interval between successive action potentials. Mean IF (MIF) was calculated for baseline and the thrust phase. The change in MIF (ΔMIF) due to the HVLA-SM was the response measure. It was calculated by subtracting MIF during baseline from MIF during the thrust phase. All neural activity is reported in impulses per sec (imp/s).

Muscle spindle neurons can have a brief, very high frequency burst of activity at the beginning of muscle movement when the spindle apparatus begins to stretch. This activity represents a response to the movement's acceleration [35]. We were interested in the spindle's response during the constant rate of thrust. Therefore MIF calculated during thrust phase did not include the first 12.5 ms which provided adequate conduction time to ensure that action potentials due to the acceleration were not included in the calculation.

### 2.10. Statistical Analysis

Sample size calculations were obtained by estimating standard deviations from two previous studies [10, 11] in which thrust amplitudes were standardized based upon body weight and displacement. Standard deviations varied between 62 and 67 imp/s. Assuming similar patterns of activity would be seen as in these previous studies cohort sizes of 20 cats would yield >99% power for the overall *F*-test and at least 80% power to detect mean differences of 60 imp/s or more between adjacent levels of thrust duration. Thus all cohorts consisted of 20 cats except the 25% BW cohort. This was the last cohort studied. With data analyses already completed for the 55% BW and 85% BW cohorts, we saw little difference in mean ΔMIF between either of these cohorts and the 25% BW cohort (as seen in Figure 4). It was deemed appropriate to reduce the number of cats.

Neural responses were compared across the 7 levels of thrust duration (25, 50, 75, 100, 150, 200, 250 ms) using a one-way ANOVA for the randomized complete block design. Each cat served as a blocking factor in order to control for the relative levels of spindle activity and intra-animal variability. An overall *F*-test was used to test whether the means were the same over the thrust durations. For statistically significant overall *F*-tests, only 6 preplanned contrasts between mean ΔMIFs at adjacent durations were compared to detect the possibility of a threshold effect. Overall *F*-tests and preplanned contrasts were tested at the 0.05 level. Data are reported as means and 95% confidence intervals (lower, upper 95% CI) unless otherwise noted. 

## 3. Results

### 3.1. Neuronal Classification

One hundred twelve lumbar paraspinal muscle spindle neurons were studied in the 6 cohorts.  Table 1 shows the distribution of classification characteristics and responses among the 6 cohorts. Each neuron's receptive field was localized to either the longissimus or multifidus muscles except for one neuron in the 1 mm cohort whose neural activity was lost before its receptive field could be localized to a specific back muscle. Lumbar longissimus muscle contained the receptive fields of 92 neurons. In this muscle, the most sensitive portion of each neuron's receptive field was most often located at the level of the L_6-7_ facet joint (34%) or the L_7_ spinous process (31%). The most sensitive portion of the remaining fields in the longissimus muscle were at the level of the L_6_ spinous process (17%), between the L_6_ and L_7_ spinous (8%), or at the level of the L_7_-S_1_ facet joint (10%). The lumbar multifidus muscle contained the receptive fields of the remaining 19 neurons. In the multifidus muscle, the most sensitive portion each receptive field was located most often at the level of L_7_-S_1_ facet joint (47%) or at the level of L_7_ spinous process (32%). The remaining fields were located at the level of the L_6_ spinous process (11%), in the multifidus between the L_6_ and L_7_ spinous (5%) or at the level of the L_6-7_ facet joint (5%).

Succinylcholine injection (ia) induced a relatively high frequency and long-lasting discharge in all 112 neurons. On average, mean maximal instantaneous discharge frequency following injection reached 57 imp/s above baseline (range: 3.3–175.9 imp/s) and remained above baseline for at least 40 s. The increase in discharge began within 15 s of injection on average (range 2.7 to 53.5 s) and became maximal within 55 s (range 6.3–183.4 s). Nearly all neurons (*n* = 101) responded to a single bolus injection of succinylcholine (100 ug/kg, ia). Eleven neurons responded following a second bolus injection. The spindle with the smallest response to succinylcholine (3.3 imp/s) had limited vascular accessibility in that the direct depolarizing effect of 0.2 mL potassium chloride (1000-fold dilution from a saturated solution) injected intra-arterially increased blood pressure but did not further increase spindle discharge. All neurons tested with vibration (*n* = 104) produced a sustained response to the vibratory stimulus. All neurons tested with bipolar muscle stimulation (amplitude: 0.1–0.3 mA; duration: 50 *μ*s, *n* = 99) were silenced by it.

### 3.2. Responses to Delivery of the HVLA-SM under Displacement Control

#### 3.2.1. Effect of Varying Amplitude of Thrust Displacement

**Figure 3:**
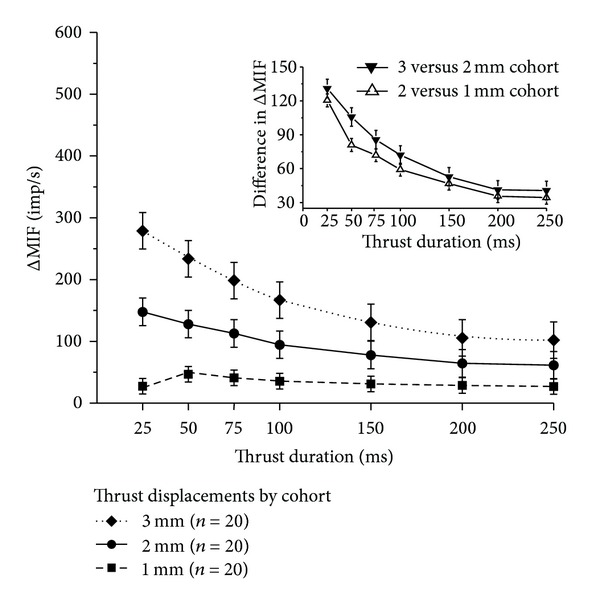
Average change in spindle discharge to 3 thrust displacements and 7 thrust durations applied under displacement control. The inset shows that lumbar muscle spindles responded more as thrust displacement increased from 2 to 3 mm than as it increased from 1 to 2 mm. Symbols represent average for the cohort. Error bars represent adjusted 95% confidence intervals. ΔMIF change in mean instantaneous frequency, imp/s = number of impulses per second. *n* = number of cats in the cohort.

As thrust displacement was increased the magnitude of lumbar muscle spindle discharge increased (Figure 3). The increases occurred at nearly every thrust duration indicated by the nonoverlapping 95% confidence intervals. Lumbar muscle spindles responded more as thrust displacement increased from 2 to 3 mm than as it increased from 1 to 2 mm because muscle spindle discharge was consistently higher at each thrust duration (inset in Figure 3).

#### 3.2.2. Effect of Varying Thrust Duration

The magnitude of thrust duration shown in Figure 3 significantly affected muscle spindle discharge for thrust amplitudes of 2 mm (*F*_6,114_ = 8.62, *P* < 0.001) and 3 mm (*F*_6,114_ = 20.22, *P* < 0.001) but not 1 mm (*F*_6,114_ = 1.41, *P* = 0.22). A pattern is clearly evident where shorter thrust durations caused graded increases in spindle discharge. However, *a priori* planned comparisons between contiguous thrust durations for the 2 and 3 mm peak thrust displacements showed that none of the increases were statistically significant suggesting there was no clear threshold value for thrust duration at which the HVLA-SM could be considered to have increased muscle spindle discharge significantly. Between the longest thrust durations of 200 and 250 ms at both the 2 and 3 mm thrust amplitudes, the change in muscle spindle discharge increased very little. However, as thrust duration became shorter than 200 ms, muscle spindle discharge increased. The steepest increase occurred when thrust duration became 100 ms or shorter. For the 3 mm thrust amplitude at thrust durations 100 ms or shorter, mean spindle discharge was generally higher than 200 imp/s, but below 200 imp/s for both the 1 and 2 mm thrust amplitudes.

### 3.3. Responses to Delivery of the HVLA-SM under Force Control

#### 3.3.1. Effect of Varying Amplitude of Thrust Force

**Figure 4:**
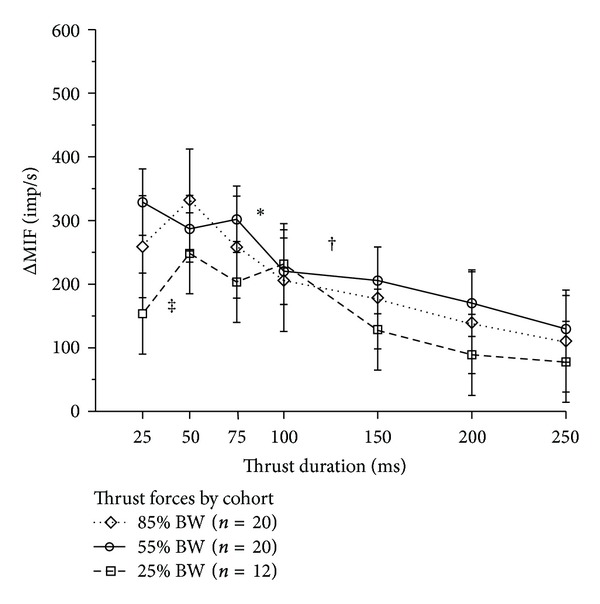
Average change in mean spindle discharge to spinal manipulation delivered under force control as thrust duration becomes shorter, thrust amplitude becomes larger. Thrust amplitude based upon each cat's body weight. Symbols represent average for the cohort. Error bars represent adjusted 95% confidence intervals. ^‡^ *P* = 0.04 between 25 and 50 ms thrust duration for 25% BW. ^†^ *P* = 0.03 between 150 and 100 ms thrust duration for 25% BW. *P = 0.03 between 100 and 75 ms thrust duration for 55% BW. Abbreviations identical to those in Figure 3.

**Figure 5:**
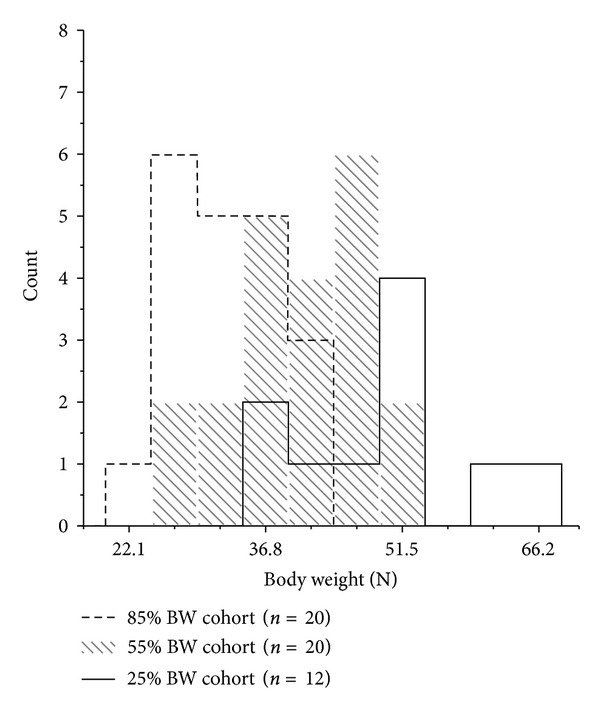
Distribution of body weights in the 3 cohorts receiving an HVLA-SM where peak thrust force was controlled. N = Newtons.

In contrast to controlling and varying the amplitude of thrust displacement, controlling and varying the amplitude of thrust force did not clearly produce graded increases in muscle spindle discharge (Figure 4). There was substantial overlap between the 95% confidence intervals across the three thrust forces. A 55% BW thrust force could increase mean spindle discharge more than the lower (25% BW) as well as the higher (85% BW) thrust force. However, it was important to recognize the possibility that some cats in the 85% BW cohort may not have received an actual thrust force (measured in Newtons) much greater than cats in either the 55% BW or 25% BW cohorts because the actual thrust force used in each of these cohorts was relative to each cat's own body weight. This is suggested in Table 1 by the fact that the mean body weight in the 85% BW cohort (32.0 N) was less than the mean body weight in the 25% BW cohort (47.5 N).  Figure 5 confirms this possibility because the distribution of body weights for the 85% BW cohort is shifted toward the left compared to the 55% BW cohort and to the right for the 25% BW cohort. For example, the cat weighing 22.1 N in the 85% BW cohort received a thrust force of 18.8 N similar to the 16. 7 N thrust force received by the cat weighing 66.6 N in the 25% BW cohort.

Consequently, to completely determine the effect of varying thrust duration under force control, we reorganized the data from the 52 cats in the three %BW cohorts. The reorganization was based upon the actual thrust force each cat received and the average body weight (38.9 N) of the 52 cats (ABW). Actual thrust force was expressed as a percentage of ABW (%ABW). The reorganization yielded 3 groups where thrust force was centered around 25%, 55%, or 85% ± 15% ABW. The 25 ± 15% ABW group received a mean thrust force of 12.1 N (range: 9.7 N to 15.2), the 55% ± 15% ABW group received a mean thrust force of 22.2 N (range: 16.0 to 26.7), and the 85% ± 15% ABW group received a mean thrust force of 30.6 N (range: 27.6 to 36.3 N). The effects of varying thrust duration based upon relative thrust force (25%, 55%, and 85% BW cohorts) and the absolute range of thrust forces (25%, 55%, or 85% ± 15% ABW reorganized groupings) are presented in the next subsection.

#### 3.3.2. Effect of Varying Thrust Duration

Thrust duration significantly affected muscle spindle discharge at all three thrust forces in the cohorts (25% BW: *F*_6,66_ = 4.53, *P* < 0.001, 55% BW: *F*_6,114_ = 7.75, *P* < 0.001; and 85% BW, *F*_6,114_ = 3.62, *P* = 0.003). Pre-planned comparisons between contiguous thrust durations suggest the presence of threshold values of thrust duration for increasing muscle spindle discharge. Significantly greater increases in muscle spindle discharge occurred between the 150 and 100 ms durations at a thrust force of 25% BW (^†^ *P* = 0.03, in Figure 4) and between 100 and 75 ms at a thrust force of 55% BW (*P = 0.03, in Figure 4). With a thrust force of 25% BW, spindle discharge during the 25 ms thrust duration was significantly less than during the 50 ms thrust duration (^‡^ *P* = 0.04, in Figure 4).

Similarly, thrust duration significantly affected muscle spindle discharge in all 3 groups reorganized based upon % ABW (25% ± 15% ABW: *F*_6,66_ = 3.77, *P* = 0.003; 55% ± 15% ABW: *F*_6,144_ = 9.46, *P* < 0.001; and 85% ± 15% ABW, *F*_6,84_ = 2.76, *P* = 0.017). Pre-planned comparisons between contiguous thrust durations also showed a significantly greater increase in muscle spindle discharge between the 150 and 100 ms durations at a thrust force of 25% ABW (*P* = 0.03). The difference between 100 and 75 ms durations at 55% ABW was not statistically significant (*P* = 0.11) but showed the steepest increase in discharge compared to the other contiguous durations. The data suggest that threshold values for thrust duration being able to evoke a significantly large increase in muscle spindle discharge depended upon delivering the HVLA-SM under linear control of force.

### 3.4. Effect of Specifically Controlling Thrust Displacement versus Thrust Force during Delivery of an HVLA-SM

Similar displacements applied to different spines do not necessarily produce the same force, and vice versa. The relationship between the two is determined by the spine's stiffness. In the present study, where the magnitudes of thrust force or thrust displacement among cats within a cohort were identical, the consequent displacement or force, respectively, could be different within the cohort. The implication for data interpretation is that conclusions based upon the type of thrust control may actually be attributable to either the consequent thrust force or displacement that was not controlled.

**Figure 6:**
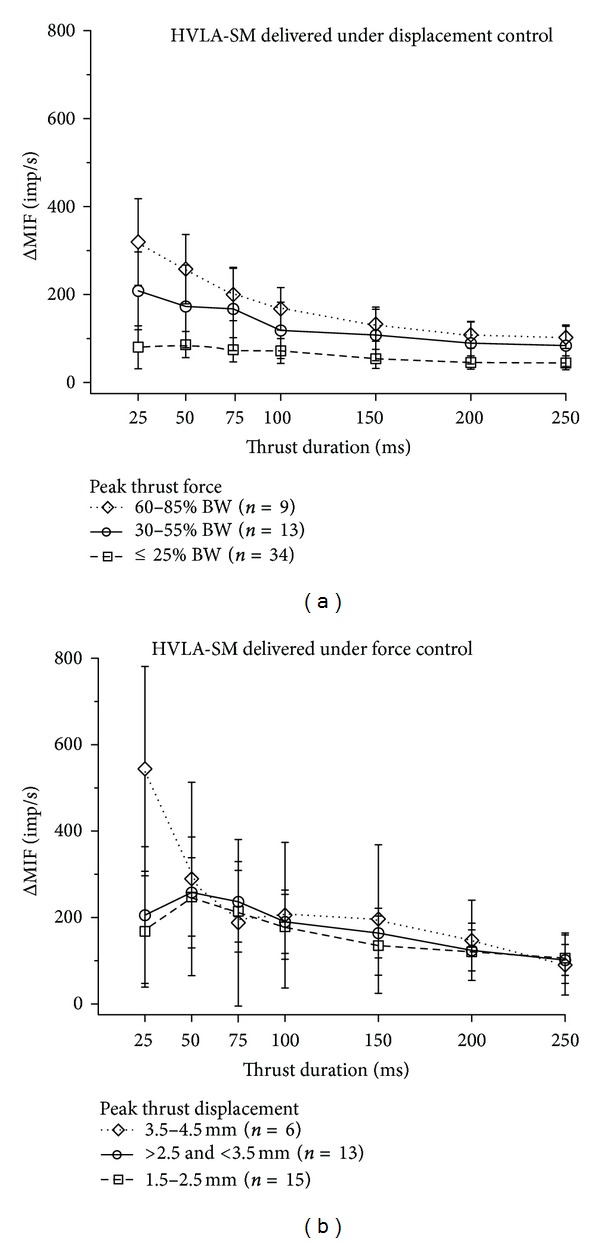
Regrouping of data shown in Figures 3 and 4 based upon (a) 3 ranges of thrust force that developed during displacement control of the HVLA-SM's delivery and (b) 3 ranges of thrust displacement that developed during force control of the HVLA-SM's. Symbols represent average for the cohort. Error bars represent adjusted 95% confidence intervals. Abbreviations identical to those in Figure 3.

To address this possibility, we reorganized the data from the 60 cats in the three displacement cohorts (shown in Figure 3). The reorganization was based upon ranges of thrust force that developed. The ranges were made to encompass magnitudes similar to the thrust force used in the force cohorts. Three groups were formed: <25% BW, 30–55% BW, and 60–85% BW. Responses from only 56 of the 60 cats fell into the 3 ranges. Thrust forces in the remaining 4 cats fell between 25% and 30% BW or between 55% and 60% BW. Data from the 56 cats are shown in Figure 6(a). A similar approach was used for the 52 cats in the three force cohorts. The reorganization was based upon the ranges of thrust displacement that developed. The ranges were made to encompass magnitudes similar to the thrust displacement used in the displacement cohorts. Three groups were formed: 1.5–2.5 mm, or >2.5 and <3.5 mm, or 3.5–4.5 mm. Thrust displacements from only 34 of these 52 cats fell into these ranges. Thrust displacements in the remaining 18 cats were either less than 1.5 mm or greater than 4.5 mm. Data from the 34 cats are shown in Figure 6(b). Under *displacement control* of the HVLA-SM, spindle responses *remained graded* with thrust amplitude regardless of whether the data were grouped according to thrust displacement or thrust force (compare Figure 3 with Figure 6(a)). Under *force control* of the HVLA-SM spindle responses were *not graded* with thrust amplitude regardless of whether the data were grouped according to thrust displacement or thrust force (compare Figure 4 with Figure 6(b)). Thus, the distinct effects of thrust amplitude on the pattern of muscle spindle discharge appeared due to the type of thrust control with which the HVLA-SM was delivered.

### 3.5. Effect of Thrust Rate

**Figure 7:**
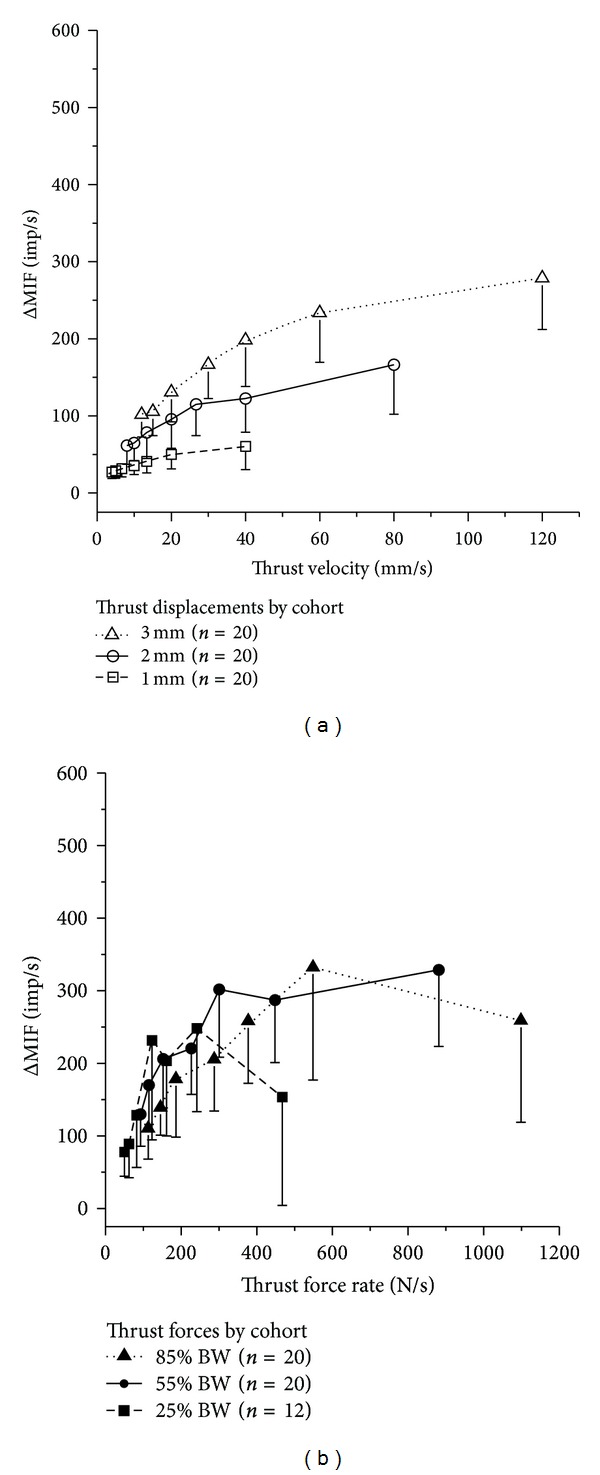
Relationship between thrust rate and the average change in mean spindle discharge. (a) Displacement control. (b) Force control. Symbols represent average for the cohort. Error bars represent adjusted 95% confidence intervals. Abbreviations identical to those in Figure 3.

Because displacement and force applied during the thrust phase of the HVLA-SM were made to increase linearly (i.e., at a constant rate), we determined how thrust rate affected the response from the muscle spindles.  Figure 7 shows the same data as in Figures 3 and 4 plotted as a function of thrust rate in each cohort. The faster thrust rates reflect the shorter thrust durations. Muscle spindle discharge tended to become maximal as thrust rate increased. This occurred at thrust velocities greater than 20–30 mm/s and at thrust force rates greater than 300 N/s.

## 4. Discussion

In the current study, we undertook an investigation using an established animal preparation as an initial step toward identifying biomechanical characteristics of an HVLA-SM that can help define qualities that contribute to its clinically effective dosage. Identification of these characteristics was accomplished by systematically varying aspects of the HVLA-SM's amplitude and duration and determining their effects upon changes in neural activity from muscle spindles in the vertebral column. While the mechanism of HVLA-SM's action remains elusive, spinal manipulation's influence on this proprioceptor, the muscle spindle, has long been thought to be an important mediator of its clinical effects [18]. This study revealed several biomechanical characteristics of the short-lever HVLA-SM that would be expected to be particularly influential on neural activity evoked during an HVLA-SM.

First, controlling thrust force and its amplitude affected the pattern of neural activity differently from controlling thrust displacement. A linear increase in thrust force produced similar muscle spindle responses regardless of the peak thrust amplitude (measured as displacement in Figure 4 or force in Figure 7(b)). On the other hand, a linear increase in thrust displacement produced spindle responses that were graded with the peak thrust amplitude (Figures 3 and 7(a)). In addition, the variability in neural discharge was less when the linear increase in thrust displacement was controlled (compare Figure 3 with Figure 4).

An explanation for these differences may lie in the spine's viscoelastic behavior because the relationship between the forces that develop in the tissues and resulting tissue displacements is nonlinear [36]. Muscle spindles lie in parallel with muscle fibers and are considered physiological length detectors. During the linear increase in thrust displacement, the HVLA-SM likely produced changes in muscle length that were linearly proportional and graded with the different magnitudes of thrust displacement. However, when the HVLA-SM's thrust was applied with a linear increase force, paraspinal muscle length would not be expected to increase linearly. The system acted as if the three different magnitudes of applied peak thrust force (25%, 55%, and 85% BW) could each produce nearly the same change in paraspinal muscle length. This behavior suggests that the thrust of an HVLA-SM delivered under linear control of force produces a more robust response from muscle spindles because their response depends little on the actual thrust amplitude.

Second, the present study showed that thrust duration and thrust rate are characteristics that affect the magnitude and pattern of proprioceptive input during the HVLA-SM. Larger changes in spindle discharge occurred as thrust durations became shorter than 150 ms (Figures 3 and 4). Thrust duration made a particularly large contribution to the neural response when the HVLA-SM was given under force control as evidenced by muscle spindle discharge significantly increasing at thrust durations of 75 to 100 ms relative to the immediately longer durations (see Figure 4). Faster thrusts under both force and displacement control do not continuously produce larger changes in spindle discharge but instead plateau becoming relatively constant at the three fastest thrust rates (Figures 7(a) and 7(b)). Combined, these temporal aspects of the HVLA-SM indicate the presence of a threshold range of thrust durations and thrust rates that lead to a stable, high frequency neural input from these muscle proprioceptors. We speculate that to be clinically effective, the velocity of an HVLA-SM needs to reach a critical value to engender an appropriate neural response. The presence of such a value or range of values needs to be established with clinical studies.

In treating patients with musculoskeletal complaints, it is not known whether clinicians attempt to control the force or displacement they apply during HVLA-SMs or even if these parameters can be controlled within specific tolerances. From a motor control perspective, the manipulative thrust can be considered a ballistic movement, being short in duration and having a high loading rate. It seems difficult to conceive that clinicians could adjust their motor strategy mid-thrust to change from force to displacement control. Clinicians may learn to control displacement in order to limit spinal movement or patient discomfort because they cannot predict how much resistive force the spinal column will provide. On the other hand, the palpatory procedures typically used to determine the segmental level to manipulate may provide the trained clinician with the ability to anticipate the manipulative force needed and initiate a motor program based upon force control. Efforts have been underway (reviewed in [7]) to understand factors that contribute to the acquisition of spinal manipulation as a motor skill [37, 38] while various techniques have been tested to train clinician's to control the biomechanical parameters that characterize a spinal manipulation [39–41].

Although the clinical consequences of muscle spindle activity evoked by a spinal manipulation cannot be determined from the present study, our results may explain a neurophysiological response reported in previous human studies where HVLA-SM causes a short-term reduction in the tibial nerve H-reflex [42–44]. While mobilizations, which are more slowly applied compared to HVLA-SM, have a similar effect, the magnitude of the reduction tends to be less while massage has no effect on the H-reflex [45, 46]. Reductions in the H-reflex indicate that the muscle spindle's efficacy for increasing alpha-motoneuron excitability has been reduced. It is known that increasing levels of muscle spindle activity reduce the H-reflex [47]. Synaptic, as well as pre- and postsynaptic mechanisms that reduce alpha-motoneuron activity, are activated by bursting activity from muscle spindle neurons [48, 49]. The higher spindle discharge frequencies found in the current study as thrust durations became similar to that used clinically (~150 ms [24–26]) may have caused the reported reductions in the H-reflex.

Several aspects of our study may moderate our conclusions regarding the biomechanical parameters of HVLA-SM that influence muscle spindle responses. Spinal manipulations are typically delivered at a vertebra's end range of motion which distract, gap, and potentially cavitate a facet joint [4, 30, 50, 51]. We did not bring the manipulated vertebra (L_6_) to its end range of motion because the magnitude of this movement would have stretched the nerve filament and tore it from the recording electrode. In addition, while nearly all manually delivered HVLA-SMs involve a posterior-to-anterior thrust component, many also involve some degree of vertebral rotation which was not taken into account in this preparation but may be added to future investigations of this nature. Finally, paraspinal tissues are also innervated by low- and high-threshold mechanoreceptors other than muscle spindles that likely respond to spinal manipulation [52]. Whether similar conclusions would arise from these receptors also requires further investigation.

## 5. Conclusions

In conclusion, we have identified several biomechanical characteristics of a lumbar HVLA-SM in an animal preparation that differentially affected the magnitude and pattern of activity from a lumbar muscle proprioceptor. These characteristics include (1) thrust amplitude and whether the peak thrust force or peak thrust displacement is increased linearly; (2) thrust duration; and consequently (3) thrust rate. The range of values which modulated the neurophysiological responses in this study may be different from values in humans. The translational usefulness of this type of animal study is its ability to provide a basis for identifying biomechanical characteristics that could be most relevant to measure and report in clinical efficacy studies of spinal manipulation. This will help the field define and ultimately identify effective dosages of spinal manipulation.

## Figures and Tables

**Table 1:** Distribution of classification characteristics.

	Muscle	COHORT (based upon thrust amplitude)						
		1 mm	2 mm	3 mm	25% BW	55% BW	85% BW	Total
Body weight mean (*N*) (SD)		38.3 (5.5)	39.1 (8.4)	39.2 (9.2)	47.5 (9.2)	40.6 (6.9)	32.0 (5.3)	38.9 (8.3)
Receptive field location (*n*)	Long. Multifidus	16	17	15	9	16	19	92
		3	3	5	3	4	1	19
Tested & responded to succinylcholine (*n*)		20	20	20	12	20	20	112
Tested & responded to vibration (*n*)		15	19	20	11	19	20	104
Tested & responded to twitch (*n*)		14	19	19	11	19	18	99

Long.: Longissimus; *N*: Newtons, SD: standard deviation, BW: body weight, *n*: number of occurrences.
